# Stapedotomy Utilizing a Fixed-Size Prosthesis in a Tertiary Care Center: A Retrospective Study

**DOI:** 10.7759/cureus.64802

**Published:** 2024-07-18

**Authors:** Rani Hammoud, Aisha Larem, Fatima Emam, Ahmad R Al-Qudimat, Adham Aljariri, Abdulsalam Alqahtani, Aya Elderee, Ali Al saadi, Amr Elhakeem, Hassanin Abdulkarim, Hassan Haidar

**Affiliations:** 1 Department of Otolaryngology - Head and Neck Surgery, Hamad Medical Corporation, Doha, QAT; 2 Department of Radiology, Hamad Medical Corporation, Doha, QAT; 3 Department of Public Health, Qatar University, Doha, QAT; 4 Surgical Research Section, Department of Surgery, Hamad Medical Corporation, Doha, QAT; 5 Department of Otolaryngology, Hamad General Hospital, Doha, QAT

**Keywords:** stapedectomy, standard-size stapes prosthesis., stapedotomy, stapes surgery, otosclerosis

## Abstract

Background

Stapes surgery success depends on several factors, including the length of the prosthesis used. Whether to use a standard-size prosthesis or measure the length of the stapes prosthesis has been debated in the literature. This study aims to assess the surgical outcomes of a stapedotomy using the standard 4.5 mm prosthesis without custom measurements.

Methodology

This retrospective study involved patients with otosclerosis who underwent primary stapedotomy using a standardized 4.5 mm fixed-length prosthesis between January 2017 and February 2023 at a tertiary care center.

Results

Out of 111 charts reviewed, 99 ears (56 males and 43 females) were studied. The mean air-bone gap (ABG) significantly improved from 27.9 ± 9.12 dB preoperatively to 3.95 ± 3.54 dB post-operatively (p-value < 0.05). Hearing results showed that out of 99 ears, 96.96% had a postoperative ABG of ≤10 dB and 98.98% ≤20 dB. Only three patients showed postoperative mild transient dizziness that lasted a few days. None of the patients had persistent dizziness for more than one week. One patient developed postoperative reparative granuloma with tinnitus and sensory-neural hearing loss. None had a recurrence of the conductive hearing loss during the study period.

Conclusion

Our retrospective study on stapes surgery utilizing a standardized 4.5 mm prosthesis without custom measurements showed notable surgical success and safety. Using a standard-size prosthesis shortens the surgical time and eliminates the complexities associated with intraoperative measurements, potentially reducing the risk of complications.

## Introduction

Otosclerosis is a hereditary condition affecting approximately 10% of the Caucasian population and is characterized by the resorption and abnormal bone deposition in the endochondrium of the optic capsule [1]. Patients with otosclerosis complain of progressive conductive hearing loss as the stapes footplate becomes more fixed over time [2]. Less frequently, depending on the extent of the disease, it can cause mixed or sensory neural hearing loss (SNHL) [3]. Stapes surgery has been the mainstay treatment for otosclerosis, showing improved short- and long-term hearing [4]. Stapes surgery is a well-established procedure that involves removing part or all the stapes bone and replacing it with a prosthesis to restore sound transmission to the inner ear [4]. The procedure’s success depends on several factors, including the extent of the disease, surgical technique, and type of prosthesis used [1,5]. The length of the stapes prosthesis has been a topic of debate in the literature, as it may influence hearing outcomes [5,6]. Some centers use fixed-length prostheses, and others use custom-measured prostheses [7]. Intraoperative prosthesis length measurement can be time-consuming, may lead to surgeon uncertainty and hesitations, and requires unnecessary incus and oval window area manipulation, unlike fixed-size prostheses. However, a fixed-size prosthesis may lead to failure of air-bone gap (ABG) closure in cases where it is short or to vestibular irritation and fistula in cases where it is too long [6-8]. In the literature, there is a lack of consensus on whether to measure or not and on the optimal prosthesis length [9,10]. Our center utilizes a standardized 4.5 mm fixed-length prosthesis for stapes surgery. This retrospective descriptive study aims to assess the surgical outcomes of stapes surgery using the 4.5 mm prosthesis without custom measurements and evaluate its efficacy and safety in patients with otosclerosis.

## Materials and methods

Data were retrospectively reviewed and collected from the medical records of all patients with otosclerosis who underwent stapedotomy between January 2017 and February 2023 at Hamad Medical Corporation, Doha, Qatar. The surgical ethical committee of the institution approved the study. We have included all patients with otosclerosis and conductive or mixed hearing loss who underwent primary stapedotomy using an unmeasured standard length 0.6 × 4.5 mm titanium soft clip prosthesis. We excluded ears with incomplete charts or lost follow-up. If both patients’ ears were eligible for inclusion, each ear was separately entered into the analysis. The data collected from the medical charts include the age, gender, nationality, height of the patient, pre- and postoperative pure tone audiograms, and postoperative dizziness or other complications.

Surgery description

All patients were operated on under general anesthesia using a trans-canal approach. After removing the stapes superstructure, a 0.8 mm calibrated footplate fenestration was made using a microdrill. Then, a 0.6 × 4.5 mm titanium soft clip prosthesis was inserted without any measurement.

The patients were discharged home on the same day, if fit. In the case of postoperative dizziness, the patient was hospitalized with complete bed rest until the dizziness was resolved. The postoperative hearing assessment was done three and six months after the surgery, with the last hearing test considered the postoperative hearing outcome.

All patients underwent a pure tone audiogram in a standard soundproof audiometric booth before and after the stapes surgery. The mean pre- and postoperative ABGs were calculated at frequencies of 0.5, 1, and 2 kHz. A postoperative ABG of 20 dB or less indicated successful surgery, and a postoperative ABG of 10 dB indicated excellent results.

Preoperative and postoperative mean bone conduction (BC) levels were calculated at 1, 2, and 4 kHz. Postoperative SNHL was defined as a deterioration in BC of more than 10 dB.

Statistical analysis

All data were collected using Microsoft Excel (Microsoft Corporation, Redmond, Washington, United States) and analyzed using STATA software (version 17, StataCorp LLC, College Station, Texas, United States). Mean differences between the two groups were assessed using t-tests, with significance determined by a p-value of <0.05. Additionally, binary logistic regression analysis was conducted, with associations considered significant if the p-value was <0.05.

## Results

Out of 111 ear charts reviewed, 99 fulfilled the criteria and were included in this study. A total of 12 ear cases were excluded due to incomplete charts or lost follow-up. This study included 85 patients (99 ears), with 14 patients having both ears operated on.

Patient’s demographic information

There were 56 (56.56%) males and 43 (43.43%) females, with a male-to-female ratio of 1.3:1. The mean age of the patients was 41.49 ± 8.57 years. The study included patients of various nationalities, with the majority being from Asia. Specifically, 73.7% of the patients were from Asia, 23.2% were from Africa, and 3% were from Europe.

Comparing pre- and postoperative hearing results

The paired t-test was conducted to compare the preoperative and postoperative ABG levels. The mean difference between preoperative and postoperative ABG levels was found to be 24.02 dB. The t-value obtained was 29.10, with a 95% CI for the mean difference ranging from 26.15 to 29.8. The p-value obtained was less than 0.001, indicating a statistically significant difference between preoperative and postoperative ABG levels. These results suggest a significant decrease in ABG levels from preoperative to postoperative conditions.

The statistical analysis revealed a significant difference between preoperative and postoperative pure-tone averages (PTAs). Postoperative PTA (mean = 26.9, SD = 8.56) showed a substantial decrease compared to preoperative PTA (mean = 54.6, SD = 10.04), with a mean difference of 24.71 dB (t = 28.02, 95% CI: 28.02-52.6). This difference was highly significant (p < 0.001). Additionally, statistical analysis reveals a significant difference in BC scores before and after the procedure. Preoperatively, the mean BC score was 29.9 dB (SD = 0.92), whereas postoperatively, it decreased to 23.8 dB (SD = 0.87). The mean difference between preoperative and postoperative BC scores was 6.06 dB (t = 7.26, 95% CI (28.1-31.7 for preoperative BC; 22.1-25.6 for postoperative BC)), with a p-value of <0.001, indicating statistical significance (Table 1).

**Table 1:** Pre- and postoperative mean hearing results Analysis using t-test, * p <0.05 ABG: air-bone gap; BC: bone conduction; PTA: pure tone average

Measures	Mean ± SD (pre-op)	Mean ± SD (post-op)	Mean difference	t	95% CI (pre-op)	95% CI (post-op)	p-value
ABG	27.9 ± 9.12 dB	3.95 ± 3.54 dB	24.02 dB	29.1	26.15-29.8	3.24-4.66	<0.001*
PTA	54.6 ± 10.04 dB	26.9 ± 8.56 dB	24.71 dB	28.02	52.6-56.6	25.2-28.6	<0.001*
BC	29.9 ± 0.92 dB	23.8 ± 0.87 dB	6.06 dB	7.26	28.1-31.7	22.1-25.6	<0.001*

**Figure 1:**
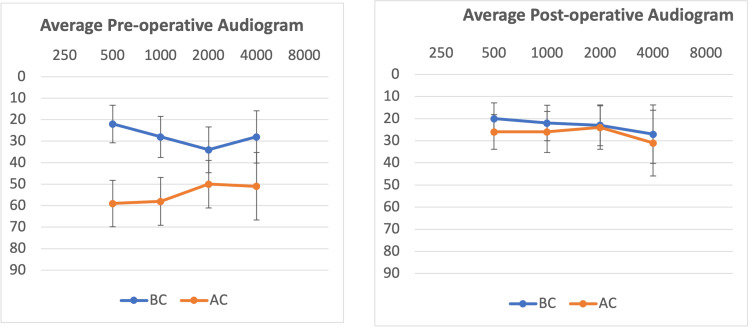
Pure tone audiogram Left chart: preoperative average pure tone audiogram; right chart: postoperative average pure tone audiogram x-axis: frequency in Hz; y-axis: hearing level in dB AC: air conduction; BC: bone conduction

Figure 1 presents the average pre- and postoperative pure tone audiograms. The average preoperative pure tone audiogram highlights the conductive hearing loss with ABG and the characteristic Carhart notch at 2,000 Hz. The average postoperative pure tone audiogram shows the ABG closure and the resolution of the Carhart notch.

**Figure 2:**
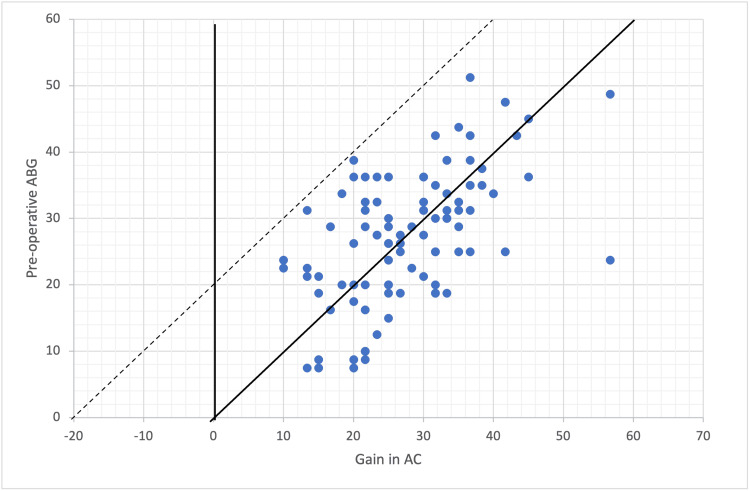
Audiometric results visualized with the AHEP, with preoperative ABG plotted against gain in AC The solid diagonal line indicates the total closure of the ABG. The area in between the solid and dotted diagonal lines marks ABG closure at ≤20 dB. Every point under the solid diagonal line is defined as an overclosure. An unsuccessful operation result regarding AC is a negative change in AC or a change in AC that was not enough to close the gap between postoperative AC and preoperative BC to 20 dB or less. This is indicated by the area above the dotted diagonal line. ABG: air-bone gap; AC: air conduction; AHEP: Amsterdam Hearing Evaluation Plot

**Figure 3:**
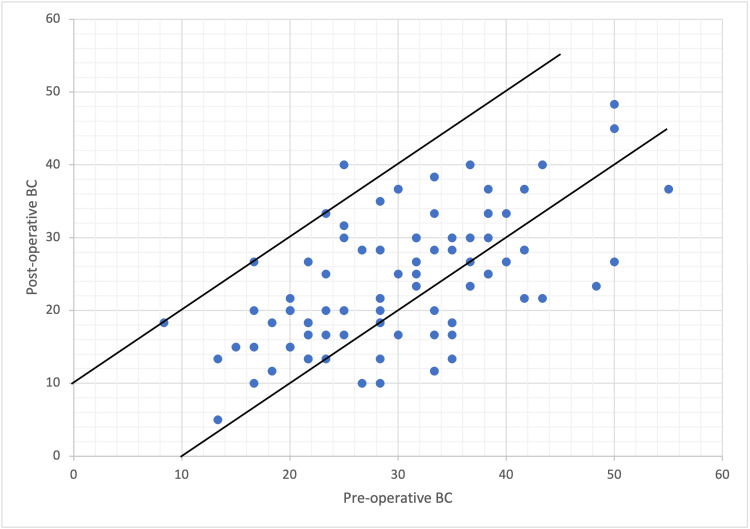
Audiometric results visualized with the AHEP, with postoperative BC plotted against preoperative BC Two diagonal lines enclose the area within BC that did not change for more than 10 dB. The area under the lower diagonal line indicates improvement in the BC. The area above the upper diagonal line indicates SNHL of more than 10 dB. AHEP: Amsterdam Hearing Evaluation Plot; BC, bone conduction

The audiometric results using the Amsterdam Hearing Evaluation Plot (AHEP) in Figure 2 show postoperative air conduction (AC) gain against preoperative ABG. All points fall below the dotted diagonal line, indicating successful ABG closure postoperatively, with some points even falling below the solid line of complete closure, indicating overclosure. Conversely, in Figure 3, which evaluates preoperative BC against postoperative BC, most cases exhibit improvement, as evidenced by their positioning below the upper diagonal line. However, one ear exceeds the upper diagonal line, suggesting a significant deterioration in BC, indicative of SNHL.

A binary logistic regression analysis was done to examine the relationship between the pre- and postoperative hearing results and both gender and height variables. The analysis shows that the male gender is significantly associated with higher odds of preoperative ABG (OR 52.77, 95% CI 1.43-1939.1, p = 0.031) compared to the female gender. However, for pre- and postoperative PTA, BC, and postoperative ABG, gender does not show a significant association. Additionally, the analysis suggests no significant association between the two height groups (<165 cm vs. ≥165 cm) and wither preoperative or postoperative ABG, PTA, or BC (Table 2, Table 3).

**Table 2:** Binary logistic regression of gender with pre- and postoperative hearing results Analysis using t-test, * p < 0.05 ABG: air-bone gap; BC: bone conduction; PTA: pure tone average

	Gender		
	Female (OR, 95% CI)	Male (OR, 95% CI)	p-value
Preoperative			
ABG	1	52.77 (1.43-1939.1)	0.031*
PTA	1	3.66 (0.06-211.5)	0.52
BC	1	0.24 (0.006-9.69)	0.44
Postoperative			
ABG	1	1.8 (0.44-7.76)	0.17
PTA	1	0.09 (0.003-2.88)	0.39
BC	1	0.04 (0.001-1.56)	0.087

**Table 3:** Binary logistic regression of height with pre- and postoperative hearing results Analysis using t-test, * p < 0.05 ABG: air-bone gap; BC: bone conduction; PTA: pure tone average

	Height		
	<165 cm (OR, 95% CI)	≥165 cm (OR, 95% CI)	p-value
Preoperative			
ABG	1	1.28 (0.32-50.54)	0.89
PTA	1	2.09 (0.036-118.4)	0.71
BC	1	5.02 (0.12-196.6)	0.38
Postoperative			
ABG	1	2.07 (0.50-8.57)	0.3
PTA	1	0.45 (0.014-14.13)	0.65
BC	1	0.15 (0.004-4.91)	0.28

Surgery success

Of the 99 ears, 98 (98.98%) showed a postoperative ABG of less than 20 dB, and 96 (96.96%) showed a postoperative ABG of less than 10 dB. Regarding BC level, the mean preoperative and postoperative BC are 29.93 ± 9.15 dB and 23.80 ± 8.60 dB, respectively, with statistical significance (p-value < 0.05) and a mean BC gain of 6.12 ± 8.11 dB.

Complications

In regard to the prevalence of postoperative complications, none of the patients had dizziness that lasted more than one week; only three patients had transient postoperative vertigo that lasted a few days. None of the patients needed revision surgery during the study period except one who developed postoperative reparative granuloma with tinnitus and SNHL of 15 dB drop in BC level. No patients developed facial palsy, tympanic membrane perforation, or had a recurrence of conductive hearing loss during the period of our study.

## Discussion

The evolution of modern otosclerosis surgery has progressed through different historical periods. The fenestration era started in the early 1900s with Holmgren [11], then in the 1950s, the stapedectomy era rose with Shea, who demonstrated that the stapes could be removed and the oval window could be covered with a vein graft [12,13]. In the following years, biocompatible material was used to create an implant prosthesis for reconstructing the sound-conducting mechanism. Over time, the technique of stapedectomy gained widespread acceptance and has undergone various stages of improvement. In the 1970s, Myers introduced the concept of stapedotomy, which involved creating a small hole in the footplate instead of complete or subtotal removal [11]. In the early 1980s, Perkins incorporated lasers for stapedotomy [11].

In pursuing perfection in stapes surgery, researchers have studied several factors that can affect the surgical outcome [4]. The influence of prosthesis length on hearing outcomes in stapes surgery remains controversial, with some studies showing that a longer prosthesis, such as a 4.5 or 4.75, had better hearing outcomes. In contrast, a shorted 3.25 had the worst [5,14]. On the other hand, one study showed no significant relationship between the hearing outcome and the prosthesis size [6]. The surgical outcome of a fixed versus a measured-length prosthesis has also been compared in the literature; one study found that there was no significant difference in hearing outcomes between patients who received fixed-length prostheses and those who received measured-length prostheses [15], while another study found that measured-length prostheses resulted in better hearing outcomes compared to fixed-length prostheses [6]. Accurately measuring the distance between the footplate and the incus can be difficult for even experienced surgeons and requires precise manipulation of instruments in a very small and delicate area of the ear; this process can increase the surgical time and can potentially cause over-manipulation of the incus and the oval window area. In addition, inaccurate measurement can result in inappropriate prosthesis length, causing complications such as prosthesis displacement, inner ear damage, incus dislocation, or persistent hearing loss [6,8]. A standard fixed-sized prosthesis eliminates this intraoperative hesitation and excessive manipulation at the critical oval window area and theoretically decreases potential complications. In addition, skipping intraoperative measurement decreases the surgical time and is of importance in cases operated under local anesthesia. In our study, the 4.5 × 0.6 mm titanium soft clip prosthesis was used, and we looked retrospectively at the hearing outcomes and complications.

The audiometric results, depicted using the AHEP, provide valuable insights into the procedure’s efficacy in improving hearing outcomes in our study. The data points in the plot in Figure 2 depicting the postoperative AC gain against preoperative ABG fall below the dotted diagonal line, indicating successful closure of the ABG postoperatively. Some points even fall below the solid of complete closure, indicating overclosure. Conversely, in Figure 3, when evaluating preoperative BC against postoperative BC, most cases exhibited improvement, as evidenced by their positioning below the upper diagonal line. However, one ear exceeds the upper diagonal line, suggesting a significant deterioration in BC, indicating SNHL. The surgical success of stapes surgery in which the ABG is less than 10 dB is as close as 95% in large-scale studies [16-21]. However, in smaller-scale studies, this number is slightly reduced to around 76-87% [15,19]. In agreement with previous literature, our current study had excellent postoperative surgical success, with 98 cases out of 99 (98.98%) showing a postoperative ABG of less than 20 dB and 96 out of 99 (96.96%) showing a postoperative ABG of less than 10 dB. In addition, our study demonstrated an average hearing gain of 24.71 dB in the AC threshold, which is comparable to previous studies [15,19,20]. It is worth noting that these results were achieved in a diverse population, as our study included patients from different nationalities. Furthermore, our study shows that the postoperative hearing results were comparable in both genders and height groups.

Postsurgical complications in stapes surgery are relatively rare, and they include persistent conductive hearing loss, displacement of the prosthesis, vertigo, SNHL, and facial nerve palsy [21]. Vertigo is seen in 8.5-45% of patients post-stapes surgery and typically resolves within one week [22], while persistent vertigo beyond four weeks is seen in 0.5-2.6% of cases [23]. In our study, none of the patients had dizziness that lasted more than one week; only three patients had transient postoperative vertigo that lasted a few days. Only one patient needed revision surgery during the study period; this patient developed postoperative reparative granuloma with tinnitus and a SNHL of 15 dB drop in BC level. Reparative granuloma is a rare and uncommon condition believed to be an inflammatory response to foreign bodies such as prostheses or intraoperative materials such as gel foam [24]. Regardless of the cause, the rate of SNHL in our study is within the average rate reported in the literature (0.4-3%) for primary stapes surgery [25]. No patients had a recurrence of conductive hearing loss or developed facial palsy, tympanic membrane perforation, or prosthesis displacement during the period of our study.

While our results align with the high success rates reported in large-scale studies, it is essential to acknowledge that our study has limitations; this includes the retrospective design, which may limit the ability to establish causal relationships. Additionally, the study’s reliance on a single-center experience may affect the generalizability of the findings to different surgical settings and techniques.

## Conclusions

This retrospective study on stapes surgery using a 4.5 mm prosthesis for patients with otosclerosis showed notable surgical success and safety. The choice of a 4.5 mm prosthesis can be a good option in situations where intraoperative measurements are not suitable due to intraoperative complexities or where other prosthesis sizes are unavailable. Future prospective multicenter studies may provide additional insights and further validate these findings.
